# Allelic Variation in Taste Genes Is Associated with Taste and Diet Preferences and Dental Caries

**DOI:** 10.3390/nu11071491

**Published:** 2019-06-29

**Authors:** Linda Eriksson, Anders Esberg, Simon Haworth, Pernilla Lif Holgerson, Ingegerd Johansson

**Affiliations:** 1 Department of Odontology/Section of Pedodontics, Umeå University, 901 87 Umeå, Sweden; 2 Department of Odontology/Section of Cariology, Umeå University, 901 87 Umeå, Sweden; 3 Medical Research Council Integrative Epidemiology Unit, Department of Population Health Sciences, Bristol Medical School, University of Bristol, Bristol BS8 2BN, UK; 4 Bristol Dental School, University of Bristol, Bristol BS1 2LY, UK

**Keywords:** taste perception, taste preference, taste genes, diet preference, diet selection, caries

## Abstract

Taste and diet preferences are complex and influenced by both environmental and host traits while affecting both food selection and associated health outcomes. The present study genotyped 94 single nucleotide polymorphisms (SNPs) in previously reported taste and food intake related genes and assessed associations with taste threshold (TT) and preferred intensity (PT) of sweet, sour and bitter, food preferences, habitual diet intake, and caries status in healthy young Swedish men and women (*n* = 127). Polymorphisms in the *GNAT3, SLC2A4, TAS1R1* and *TAS1R2* genes were associated with variation in TT and PT for sweet taste as well as sweet food intake. Increasing PT for sweet was associated with increasing preference and intake of sugary foods. Similarly, increasing TT for sour was associated with increasing intake of sour foods, whereas the associations between food preference/intake and TT/PT for bitter was weak in this study group. Finally, allelic variation in the *GNAT3, SLC2A2, SLC2A4, TAS1R1* and *TAS1R2* genes was associated with caries status, whereas TT, PT and food preferences were not. It was concluded that variations in taste receptor, glucose transporter and gustducin encoding genes are related to taste perception, food preference and intake as well as the sugar-dependent caries disease.

## 1. Introduction

A balanced diet is important for growth, well-being, development, the immune system and prevention of diet related diseases [1,2,3]. The driving incentives behind food preferences are not fully understood, but experience and taste perception are suggested as substantial driving forces [4,5]. Thus, dietary habits and patterns are influenced by geographical, cultural, and socio-economic factors, but also biological factors, such as genetic polymorphisms [6,7,8,9]. Humans can distinguish sweet, sour, salty, bitter and umami tastes [10] but sensitivity differs between individuals and species [4,7]. Evolutionary taste ability has been vital for identification of eatable foods. i.e., sweet taste signalling safe foods and bitter foods poisonous foods [7]. 

A taste sensation results when a chemical substance from food triggers the taste receptors in the taste buds on the fungiform papillae on the tongue [10]. Individual taste receptivity is associated with the number of fungiform papillae and taste receptor phenotype variation from coding gene polymorphisms, such as the *TAS1R2* and *TAS1R3* for sweet and *TAS2R38* for bitter taste receptors [6,7,8,9]. Other genes that are suggested as influential on taste or sugar intake are the *GNAT3* gene encoding gustducin [11] and the glucose transporter genes (*SLC2A2* and *SLC2A4*) [6,12] which are all expressed in taste cells on the tongue. The relationship between taste perception and food preferences has been studied for bitter taste [9] and to some extent sweet taste [5]. However, the number of studies on the linkage between taste gene variations and taste perception/preference combined with actual food preference/selection is limited, and, apart from a few recent genome wide scans [13,14,15], most studies target a limited number of genetic polymorphisms for one taste or taste gene [5]. 

Dental caries is a diet related disease where carbohydrates that are fermented by tooth colonizing bacteria are crucial for disease development. As signs of caries may develop in a comparably short time caries is a suitable model to evaluate the complexity between taste gene variations, taste and food preferences, and disease development. Specifically, driving forces for sweet foods are of interest since lowered pH from sugar fermentation induces tooth crystal dissolution and favours pH tolerant, caries associated bacteria [16,17]. Besides the local effects of sugars, inadequate intake of nutrients, such as protein, vitamin D, calcium, phosphate and protein, may act systemically on tooth formation and saliva production and affect disease susceptibility [16].

The aim of the present study was to assess associations between single nucleotide polymorphisms in genes reported to associate with the intake of sweet foods or sweet, bitter or sour taste perception and food preferences and intake and caries status in young Swedish men and women.

## 2. Materials and Methods

### 2.1. Study Subjects 

Men and women between 18 and 23 years of age (*n* = 127) were consecutively recruited from one public dental health care clinic in the city of Umeå, Sweden as they came for a routine examination at the dentist´s office. All participants were of European ancestry. Those who had a chronic disease, had taken antibiotics within the preceding 6 months, or declined to take part in the taste tests were not recruited. At the visit, saliva was collected, taste tests were performed and an oral examination was carried out. Information on the participant’s health status, and medication was recorded using standard questions within the dental records. Additional information on use of anti- and probiotics, health history, body weight and height, level of education, physical activity, tobacco and alcohol use and diet were obtained using supplementary questionnaires. 

### 2.2. Genotyping of Target Genes 

Whole saliva was collected into ice-chilled tubes under chewing stimulation and DNA extracted using the GenElute Bacterial genomic DNA kit (Sigma-Aldrich Co, Stockholm, Sweden). The final quality and quantity of the DNA was evaluated using a Qubit analyzer (Thermo Fisher, Wilmington, DE, USA). Following a literature search, 11 genes with a reported association with taste or dietary intake were selected for further evaluation (Table S1). The selection of SNPs was based on the literature and identification by the HAPMAP snptag program [18]. (Table S2). The SNPs selected by snptag were: (i) located in the gene of interest or within a 10.000 base pair window up- and downstream of the target gene, (ii) had minor allele frequency of 0.05 or greater and (iii) were not in perfect linkage disequlibrium (LD), with a threshold of 0.8. HAPMAP snptag was configured to use the Northern Europeans from Utah (CEU) population for LD estimation, require a minimum of 5 valid genotyping pairs for LD estimation and assume a complete LD for SNPs located more than 250,000 bases apart. A total of 94 SNPs in the *TAS1R1*, *TAS1R2*, *TAS1R3*, *TAS2R16*, *TAS2R38*, *TAS2R50*, *SLC2A2*, *SLC2A4*, *GNAT3*, *SCN1B* and *TRPV1* genes were selected and genotyped. 

Genotyping was performed at SciLife, Uppsala using the SNP&SEQ Technology Platform with a multiplexed primer extension chemistry of the iPLEX assay with detection of the incorporated allele by mass spectrometry using a MassARRAY analyzer (Agena Bioscience, Hamburg, Germany). Raw data from the mass reader was converted to genotype data using the Typer software (Agena Bioscience). One SNP marker (rs12030797 in the *TAS1R3* gene) received a call rate of 0% and was not included in further analysis leaving 93 SNPs with genotype data. None of the 93 SNPs deviated from Hardy–Weinberg equilibrium (*p* > 0.001), and they had an average call rate per sample of 99.8% and overall call rate of 99.8%. Genotyping data are uploaded at figshare [19].

### 2.3. Recording of Taste Threshold (TT) and Taste Preference (TP)

The participants tasted a series of room temperature sour, bitter and sweet solutions. Based on previous publications [20,21,22] and pilot testing among six non-participants, six concentrations ranging from a “hardly distinguishable” to a “distinct” taste were prepared. Tap water was used as a negative control. Each participant rinsed the mouth with water for one minute, and then swirled the test solutions in the mouth for approximately 10 seconds before expectorating it. The solutions were given in increasing concentrations in each taste series, and the taste series were given in the order sour, bitter and sweet. Between each concentration and each taste series, the participants rinsed the mouth with water for approximately 10 seconds. The taste threshold (TT), i.e., the concentration at which the respondent could distinguish the taste from water, and the preferred taste (PT), i.e., the concentration the respondents chose as their favourite, was recorded. For each taste series, the participants were dichotomized into a high and low group based on each of the TT and PT distributions. 

The following solutions were used: (i) ascorbic acid in tap water (sour) in the concentrations 0.0, 0.1, 0.5, 1.0, 5.0, 20.0 and 40.0 g/L; (ii) quinine hydrochloride in tap water (bitter) in the concentrations 0.0, 0.01, 0.05, 0.1, 0.2, 0.4 and 0.8 g/L; and (iii) sucrose in tap water (sweet) in the concentrations 0.0, 5.0, 15.0, 30.0, 60.0, 120.0 and 240.0 g/L. The test concentrations were referred to as TT and PT concentration levels and measures were treated on an ordinal scale (1 to 7). Stock solutions of each taste were prepared in sterile glass bottles and stored at 4 °C. Shortly before use, 10 mL diluted aliquots were dispersed into colour coded test tubes. Hence, the taste was blinded to both the test person and examiner through the colour coding, whereas the concentration order was known.

### 2.4. Recording of Food Preferences and Food Intake

Participants completed two electronic questionnaires during the clinic visit, one measuring the habitual diet intake over the latest year and one on food item preference.

Habitual diet intake was recorded with a semi-quantitative food frequency questionnaire including 93 food items/food aggregates designed to capture a range of common Swedish foods. Intakes were reported on an increasing, nine-level scale, including never, less than once a month, 1–3 times per month, once a week, 2–3 times a week, 4–6 times a week, once a day, 2–3 times a day, and 4 or more times a day. Portion sizes were estimated as standard portions, i.e., an egg, or from photographs showing four portion sizes of staple foods (potatoes, rice, pasta, etc.), meat/fish and vegetables. Missing values were imputed with median values from a large database on diet intake in the target population [20]. Intake frequencies were calculated, and energy and nutrient intakes were estimated by multiplying intake frequencies by portion sizes and the energy/nutrient contents in the food composition database at the National Food Administration [23]. Estimated intakes of energy, nutrients, vitamins and minerals were validated against repeated 24 h dietary records and/or biomarkers [24,25,26,27]. In addition, the 93 reported food items/food aggregates were categorized into sour, bitter, or sweet or neutral taste categories and intake frequencies were estimated for each group. 

Food preferences (as a proxy for taste preference), were recorded on a 6-level scale (with a 7th option for “Do not know”) [12]. The respondents were presented with images of 26 different food items selected to represent sour, bitter, sweet or neutral taste. They were requested to report their personal liking of each food on a scale graded “love”, “like”, “it is ok”, “not so good”, “dislike”, “hate” and “do not know”. The options were spelled out and illustrated by a face icon. The scores for foods representing the different tastes were summarized into a score for each taste. “Do not know” answers were treated as missing values and excluded from statistical analysis.

### 2.5. Caries Scoring

Tooth surfaces (S) with caries in the enamel (e) or into the dentine (D), had a filling (F), or were missing (M) were recorded from visual and radiographic examinations. Caries in the enamel (e) was scored based on a colour change on visual examination, palpable lesion using a dental probe or signs of initial demineralization on bitewing radiographs. Caries in the dentine (D) was scored when a cavity was present or when demineralization extended into the dentine on bitewing radiographs. The total number of decayed and filled tooth surfaces (DeFS, caries experience) was calculated. The M component was not considered if tooth loss occurred for orthodontic reasons or severe hypomineralization in this study group. 

### 2.6. Statistical Analyses

Discrete measures are presented as percentages in groups and differences in distribution between groups were tested with a Chi^2^ test. Normally distributed variables were presented as means with 95% confidence interval (CI) or standard error (SE), and differences between groups were tested with a parametric test. Mean values for energy and energy adjusted nutrient intakes were adjusted for sex using general linear modelling. Spearman correlation tests were used to assess correlations between variables. These analyses were performed using SPSS version 25 (IBM Corporation, Armonk, NY, USA), the tests were two-tailed and *p*-values < 0.05 were considered statistically significant. 

Haploview software (version 4.2) was used to evaluate characteristics of SNPs, potential haploblocks [28] and associations between genetic variation (SNPs and haploblocks) and phenotypes. 

Multivariate generalized linear modelling was used to assess the associations between each of the SNPs (predictors) and the TT or PT concentration levels (categorical (ordinal) measures), food preference scores or reported food intakes (both continuous measures) as dependent variables. Sexes were included as covariates to improve model fit. Similar models were run for caries but with tooth brushing as a covariate. The default method in Haploview, i.e., odds ratios (OR) with 95% CIs calculated from Chi^2^ tests was used for association analyses between haploblock variants and taste and food related phenotypes. To create dichotomous outcomes for use in these analyses, TT/PT traits were dichotomized based on their distribution in the study group using the rank-split 2 group function in SPSS. Caries status was dichotomized as caries-free (DeFS = 0) or caries-affected (DeFS >0). All tests were two-tailed and the Benjamini and Hochberg procedure applied to reduce the risk of type I errors due to multiple testing. Thus, only p-values smaller than a Benjamini and Hochberg false discovery rate of 0.05 were considered statistically significant. The effects of genotypes on these outcomes were assessed in single allelic association (a versus A) models using haploview and recessive (aa versus aA + AA), dominant (aa + aA versus AA) and additive (aa > aA > AA) models using multivariate generalized linear modelling, where “a” refers to the minor allele.

Finally, Partial Least Square modelling (PLS, SIMCA 15, Sartorius Stedim Data Analytics AB, Malmö, Sweden) was used to illustrate genotype distributions among participants characterized by their reported food preference or intakes. The PLS models evaluated the TT levels for sweet taste or the daily intake of sweet foods as dependent variables (y-variable) against an independent block (x- variables) including the intake frequencies of ice-cream, candy and chocolate, sugar and honey, marmalade or jam, cookies and pastries, fruit juices and sodas and daily intakes of total carbohydrates, sucrose, disaccharides, monosaccharides, and polysaccharides, and preferred and selected sweet sour and bitter foods and tastes. Results were displayed in a score plot (the 2- dimensional projection with the maximal separation of the individuals in the data matrix). The magnitude of the y variable was illustrated by symbol size and SNP variant for each individual superimposed. Variables utilized for PLS regression were auto-scaled and logarithmically transformed as needed to improve normality.

### 2.7. Ethical Approval 

The study was approved by the Regional Ethical Review Board in Umeå, Sweden (Dnr 2012- 111- 31M and two addendums, Dnr 2015-389-32M and Dnr 2017-450-31M) and abided by the Declaration of Helsinki, including obtaining written consent from the participants, and the *General Data Protection Regulation* (GDPR). 

## 3. Results

### 3.1. Study Group Characteristics

Overall, the participants were normal weight, i.e., a mean Body Mass Index (BMI) of 23.0, close to 5% smoked and close to 9% used Swedish snus (snuff) (Table 1). Sex-adjusted mean daily energy intake was 1748 kcal/day and total carbohydrate and sucrose intake represented 40.6 and 6.0 percent of the daily energy intake (E%) (Table 1). All participants had completed the FFQ, but 5 participants had not completed the food preference questionnaire and were excluded from association analyses. The proportion of questions in the food preference questionnaire which were unanswered or had a response of “do not know” ranged from 0.0% to 8.9% with a median value of 0.8%. Sensitivity analyses where missing answers were imputed with median values were run and did not reveal any significant difference from the results where they were excluded (data presented below). 

All participants had a saliva flow rate above 1 mL/min (mean flow rate 1.5 mL/min). Signs of caries (untreated (De) and/or treated (F)) were present in 56.7% of the participants with a mean DeFS of 4.4 (95% CI 3.4, 5.6) tooth surfaces.

The characteristics of SNPs including tests for Hardy–Weinberg equilibrium, percentage of non- missing genotypes, and minor allele frequencies are presented in Table S1.

### 3.2. Taste Threshold (TT) and Preferred Taste (PT)

**Figure 1:**
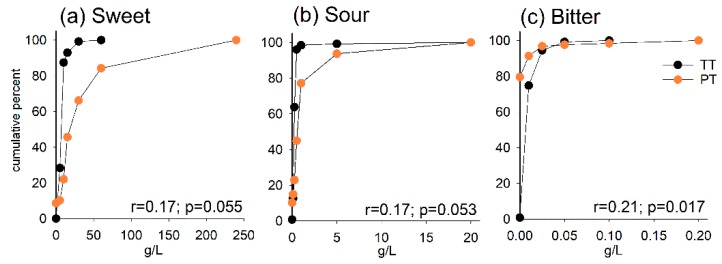
Cumulative percentage curves for taste threshold (TT, black) and preferred taste (PT, orange) by increasing test solution concentrations for (**a**) sweet, (**b**) sour and (**c**) bitter. Spearman correlation coefficients (r) are shown at the bottom of each plot.

The cumulative percentages by the concentration at which the participant distinguished a sweet, sour or bitter taste (TT_sweet_, TT_sour_, TT_bitter_) and the concentration they preferred (PT_sweet_, PT_sour_, PT_bitter_) are shown in Figure 1a–c. PT_sweet_ displayed the widest distribution, followed by PT_sour_, whereas > 85% of the participants had their PT_bitter_, TT_sweet_, TT_sour_ and TT_bitter_ concentration levels within the three lowest test concentrations. The correlations between TT_sweet_ and PT_sweet_ (r = 0.17, *p* = 0.055), TT_sour_ and PT_sour_ (r = 0.17, *p* = 0.053) and TT_bitter_ and PT_bitter_ (r = 0.21, *p* = 0.017) were all positive but modest.

Following the result that the widest distribution for individual taste preference was seen for sweet taste preference (PT_sweet_), we compared characteristics for those who preferred sweeter solutions with those who preferred less sweet solutions. Those in the former group had higher BMI and saliva flow rate, but no other difference was found (Table 1). 

### 3.3. Taste Receptor Gene Variation and Taste Threshold and Preference

**Figure 2:**
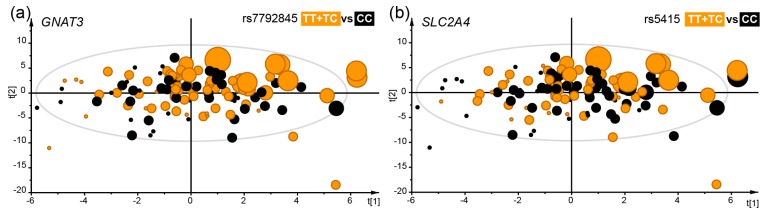
The distribution of genotypes illustrated in scatter plots from multivariate Partial Lest Square (PLS) modelling with TT_sweet_ concentration levels as the dependent variable and various sweet food intake proxies and sweet, sour and bitter foods and tastes as the independent block. The model explained (R^2^) 55% and predicted (Q^2^) 7% after validation by the “leave-one out” method. Each dot represents a participant and the size illustrates TT_sweet_ concentration levels. Participants in the *GNAT3* rs7792845 TT + TC genotype group (**a**) or the *SLC2A4* rs5415 TT + TC genotype group (**b**) both positively associated with TT_sweet_ concentration levels are shown in orange. The scatter plots are score-loading plots where the scores t(1) and t(2) are the new created variables summarizing the x-variables. The oval circle illustrates the tolerance ellipse based on Hotellings of T2, and observations located outside the ellipse indicate potential outliers.

Generalized linear modelling, including sex as covariate, identified two SNPs in the *GNAT3* gene (rs17260734, rs7792845) and two in the *SLC2A4* gene (rs2654185, rs5415) which were associated with a need for higher concentration levels to identify a sweet taste (TT_sweet_). The most prominent effect was seen for rs5415 (TT + TC vs. CC) (β = 0.49, SE = 0.14, *p* = 0.0003). One SNP in the *TAS1R1* gene (rs4908923), and one in the *TAS1R2* gene (rs9988418) were associated with a preference for lower test concentrations of sweet (PT_sweet_), whereas a different SNP in the *TAS1R2* gene (rs286552778), and also one in the *GNAT3* gene (rs7792845), had the opposite association, i.e., a preference for higher concentration levels of sweet (Table 2, Figure 2a,b). The most prominent effect on sweet preference (PT_sweet_) was for rs9988418 *TAS1R2* (TT + TC vs. CC) with β = −2.2, SE = 0.65, *p* = 0.001. 

Besides its association with a preference for less sweet tasting solutions, the rs4908932 SNP in the *TAS1R1* gene, together with rs35874116 in the *TAS1R2* gene, was associated with a preference for a sourer test solution (PT_sour_) (Table 2). In contrast, a different SNP of the *TAS1R2* gene (rs12035074), was associated with preference for less sour concentrations (PT_sour_) (β = −0.25, SE = 0.07, *p* = 0.0003) as well as an ability to recognize sour taste at low concentration levels (TT_sour_) (β = −0.13, SE = 0.04, *p* = 0.003) (Table 2). The latter association was also seen for rs6947745 in in the *GNAT3* gene. Haploview analysis suggested that the rs12035074 formed part of a haplotype within *TAS1R2* which was also tagged by rs12036097 and that the CG haploblock (rs12035074-C, rs12036097-G) was associated with a preference for less sour concentration levels, i.e., OR (95% CI) of 0.4 (0.2, 0.7), *p* = 0.001 to be in the upper versus lower dichotomous group.

For bitter taste perception (TT_bitter_), the rs2218820 (TT vs. TC + CC) allele in the *TAS2R50* gene was associated with a need for the higher concentration levels to perceive the taste (β = 0.17, SE = 0.05, *p* = 0.002) (Table 2). Haploview analysis suggested a bitter taste associated haploblock in *TAS2R16*, which was tagged by rs10772397, rs1376251 and rs6488334. The *TAS2R16* haploblock TCC (rs10772397-T, rs1376251-C and rs6488334-C) had an OR (95% CI) to be in the higher TT_bitter_ dichotomous group of 0.4 (0.2, 0.7), *p* = 0.005, but for the TTC haploblock the corresponding OR was 2.4 (1.3, 4.4), *p* = 0.004.

### 3.4. Food Preference and Food Intake in Taste Categories 

**Figure 3:**
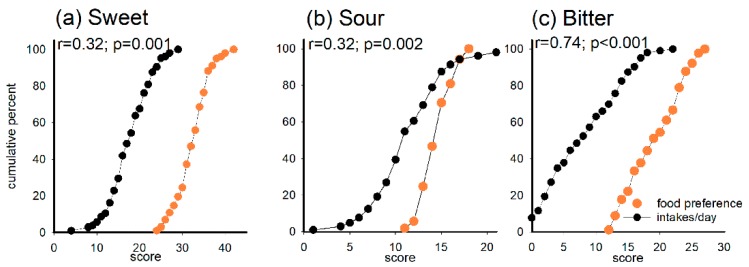
Cumulative percentage curves for food preference scores (orange) and consumption frequency (black) in the taste categories (**a**) sweet, (**b**) sour and (**c**) bitter. Spearman correlation coefficients (*r*) are shown at the top of each plot.

The cumulative percentages by preferred food scores and food consumed frequencies in the taste categories sweet, sour and bitter and correlations between food preference and food consumption are shown in Figure 3a–c. Moderate correlations were found between preference and intake of sweet foods (r = 0.32, *p* = 0.001), and sour foods (r = 0.32, *p* = 0.002), whereas preference and intake of bitter foods were strongly correlated (r = 0.74, *p* < 0.001).

### 3.5. Taste Receptor Gene Variation and Food Preference and Selection 

**Figure 4:**
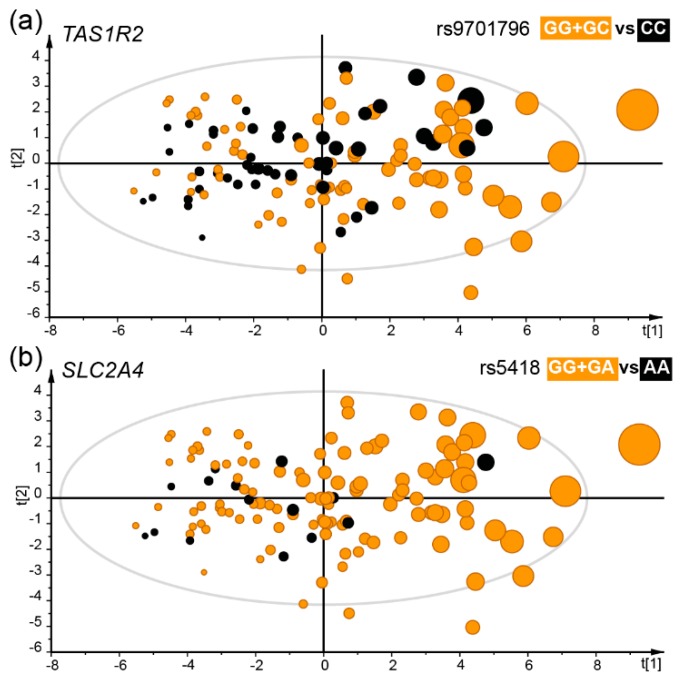
The distribution of genotypes illustrated in scatter plots from multivariate PLS modelling with daily intake of sweet foods as the dependent variable and various sweet food intake proxies and sweet, sour and bitter food and tastes as the independent block. The model explained (R^2^) 41% and predicted (Q^2^) 20% after validation by the “leave-one out” method. Each dot represents a participant and the size illustrates the number of sweet food intakes per day. Participants in the *TAS1R2* rs9701796 GG + GC genotype group (**a**), or the *SLC2A4* rs5418 GG + GA genotype group (**b**) are shown in orange. Both these genotype groups were positively associated with sweet food intake. The scatter plots are score-loading plots where the scores t(1) and t(2) are the new created variables summarizing the x-variables. The oval circle illustrates the tolerance ellipse based on Hotellings of T2, and observations located outside the ellipse indicate potential outliers.

Generalized linear modelling identified two SNP in the *TAS1R2* gene (rs9701796 and rs28374389) and two in the *SLC2A4* gene (rs5418 and rs2654185) which were associated with less intake of sweet foods (Table 2, Figure 4a,b). For *TAS1R2,* the β-value (SE) was –0.42 (0.13) servings per day, *p* = 0.003 for the rs9701796 GG + GC vs. CC genotype group and for the rs28374389 (CC<CT<TT) genotype group –0.25 (0.08) servings per day, *p* = 0.003. For *SLC2A4*, the (AA vs. CA + CC) genotype group of rs2654185 the β-value (SE) was –0.51 (0.16) servings per day, *p* = 0.002, and for the rs5418 (GG + GA vs. AA) genotype group –0.50 (0.16) servings per day, *p* = 0.002.

### 3.6. Correlations between Taste Perception and Preference and Food Preferences and Intake

Correlations between scores in the taste detection and preference tests and reported food preference and intakes in food clusters, of foods items representing the tree taste categories and some selected nutrients are presented in Table 3. A full list of all foods and food groups is shown in Table S6. The strongest positive correlations were seen between a preference for more sweet tasting test solutions (PT_sweet_) and greater liking of sweet foods as well as higher intake of sugar containing foods (r = 0.36, *p* < 0.001 and r = 0.22, *p* = 0.018, respectively). Similar correlations were seen for specific sugar containing foods, for example between PT_sweet_ and reported intake of sweet rolls (r = 0.24, *p* = 0.010), raisins (r = 0.24, *p* = 0.008), and disaccharides (r = 0.24, *p* = 0.008). In addition, a weak positive correlation was seen between increasing concentration levels for perception of sweet taste (TT_sweet_) and intake of sugar containing foods (r = 0.18, *p* = 0.048). 

“Best-liked” concentration levels of bitter (PT_bitter_) tended to correlate positively (though not statistically significant) with bitter tasting foods in general and especially with Brussel sprouts (r = 0.19, *p* = 0.044), boiled coffee (r = 0.18, *p* = 0.042) and beer (r = 0.18, *p* = 0.046), but there were also negative correlations, with preference for sour foods in general (r = −0.22, *p* = 0.019) and especially for juice (r = −0.23, *p* = 0.012). Further, a negative correlation was found between the ability to recognize bitter taste (TT_bitter_) and intake of sweet foods (r = −0.24, *p* = 0.008), syrups (r = −0.23, *p* = 0.010), pancakes (r = −0.18, *p* = 0.048), and sucrose (r = −0.22, *p* = 0.014). Requirement for higher concentrations to recognize a sour taste was negatively correlated with reported intake of sour foods (r = −0.27, *p* = 0.003), apples/pears/peaches (r = −0.29, *p* = 0.001), and intake of vitamin C (r = −0.21, *p* = 0.018). Preference for more sour tasting test solutions was positively correlated with a high preference for lemons (r = 0.20, *p* = 0.027).

### 3.7. Taste Receptor Gene Variation and Caries Status 

Generalized linear modelling, including tooth brushing as a covariate, was applied to evaluate genetic polymorphisms in the sweet taste associated genes *TAS1R1*, *TAS1R2*, *TAS1R3*, *GNAT3, SLC2A2*, *SLC2A4, SCN1B* and caries scores (DeFS).

Genetic polymorphism within the *GNAT3* gene (rs6962693) was associated with reduced caries scores with an estimated mean (95% CI) of 1.2 (0.5, 2.8) vs. 4.5 (3.5, 5.8) caries affected surfaces (DeFS) in the GG + GT vs. TT genotype group (Table 4). In contrast, variations within the *TAS1R1* (rs4908932), *TAS1R2* (rs6685177, rs28374389 and rs28410948), *SLC2A2* (rs1996220, rs5400 and rs11917504), and *SLC2A4* (rs5415) genes were associated with increased caries scores. The most prominent effect was for *SLC2A2* rs11917504 with mean (95% CI) 7.8 (5.8, 10.4) vs. 2.2 (1.3, 3.7) DeFS for the TT + TA vs. AA genotype group (Table 4). These results were consistent with those from haploview where the OR (95% CI) to have signs of caries (yes/no) was 3.9 (1.8, 8.7), *p* = 5 × 10^−4^ for those with the rs5412-A allele of the *SLC2A4* gene compared to the G allele. Haploview analysis also suggested that rs5412 formed part of a *SLC2A4* haploblock also tagged by rs5415 and rs5418. The ACA block (rs5412-A, rs5415-C and rs5418-A) was associated with higher caries prevalence (OR (95% CI) of 3.9 (1.8, 8.7), *p* = 5 × 10^−4^), whereas the GCA block was associated with lower caries prevalence (OR (95% CI) of 0.4 (0.2, 0.6) *p* = 2 × 10^−4^).

No association was found between measures of sweet taste perception or food preference/consumption and caries when evaluated in general linear modelling including sex and tooth brushing frequency as covariates.

## 4. Discussion

In the present study we genotyped allelic variation in genes reported to associate with intake of sweet foods or sweet, bitter or sour taste, and examined the effects of these genetic polymorphisms on perception and preference for sweet, sour and bitter tastes, food preferences and consumption, and caries status (a disease influenced by sugar consumption). We confirmed that genetic polymorphisms in these genes are associated with taste perception and frequency of sweet and bitter food consumption. A main finding was that gene variants tested were associated with caries status, especially the novel association with *SLC2A4*, whereas no association was found between measures of sweet taste perception or food preference/consumption and caries. 

The strengths of the study include the screening of a larger set of SNPs in taste receptor genes and other genes anticipated to associate with taste or food intake than previously, and the detailed phenotypes which included both physically-tested taste perception and preference and self-reported food preferences and intakes, as well as a diet related disease outcome. A further strength is that we consider the 127 participants to be representative for young men and women in the catchment area. This opinion is substantiated since the sampling frame was embedded in a dental clinic where the participants attended for their regular dental check-up and, because oral examination and treatment is provided free at the point of delivery to people aged 23 years or younger in Sweden, the attendance rate is very high. The consecutive enrolment and fact that all eligible participants agreed to take part seemingly limit the risk of selection bias. The weaknesses relate to the inherent difficulties in measuring diet using questionnaires, where responses may be prone to error. To help address this, two aspects of diet exposure were recorded, using a traditional semi-quantitative FFQ to estimate habitual intake and a more novel questionnaire targeting preference for foods and intake estimates was adjusted for reported energy intake [29]. Though the two instruments are designed to target different food aspects, data obtained by the latter instrument, which has been claimed to be less prone to bias [30], correlated positively with reported intakes. Though the correlations were weak, they may be seen as a potential quality support for the FFQ registered intakes. A further limitation is that the size of the study group only allowed for common allelic variation to be detected and imposed the need to choose a limited set of gene candidates for statistical power reasons. Thus, it is likely that other genes and low-frequency variation within the genes included here are also relevant, but these were not evaluated in the present study. 

Previous studies on the genetic influence on taste preference have mainly focused on sweet and bitter tastes and the *TAS1R* and *TAS2R* gene families. There are three proteins in the TAS1R family receptors, TAS1R1, TAS1R2, and TAS1R3, encoded by their respective genes, *TAS1R1*, *TAS1R2*, and *TAS1R3*. TAS1R2 and TAS1R3 form a heterodimer, which binds and responds to sugars, synthetic sweeteners, d-amino acids, and some proteins, such as miraculin [31,32]. The present study provides evidence for that gene variation within the *TAS1R2*, but not the *TAR1R3*, which is linked to both perception (TT) and preference (PT) of sweet foods. This is in line with previous studies where genetic variations within the *TAS1R2* gene were associated with the ability to taste and consumption of sugar [33], and sugar consumption in overweight and obese individuals [34]. The association between sour taste and the *TAS1R1/R2* genes was unexpected. Since we have not found any molecular explanation for this it may be speculated that sour preferences may be synergistically linked to other tastes similar to that reported for bitter taste [9].

The *GNAT3* gene, for which allelic variation was associated with TT_sweet_, PT_sweet_ and caries status in the present study, encodes the α-gustducin protein. α-Gustducin is a G-protein subunit that is expressed predominantly in taste cells [35] and has been associated with intracellular signaling cascades underlying taste transduction mainly from the TAS1R2-TAS1R3 sweet receptor. Mice lacking α-gustducin have impaired ability to register sweet taste [36,37]. Previous reports have linked *GNAT3* polymorphisms to sucrose perception and predicted that 13% of the variation in sucrose perception could be explained by *GNAT3* gene variation [11]. The findings in the present study support the hypothesis that genetic variation in *GNAT3* is associated with variation in both threshold (TT) and preferred (PT) concentrations of sweet solutions.

In the present study group, neither taste perception or preference nor food preference or intake correlated with caries status, but polymorphisms of several evaluated SNPs did. The lack of association may reflect underreporting of sugar intake, which was overcome by the use of well- measured genetic variation, or that there was little reported variation in diet intake as the study group was culturally homogenous. The finding that *TAS1R2* SNPs were associated with caries status in Swedish young men and women is in line with previous studies reporting that genetic variation in *TAS1R2* is associated with caries status in children and adults [38,39,40]. However, none of the caries associated *TAS1R2* SNPs were found to link to sweet preference or intake. Previously, higher caries scores have also been associated with carriers of the *SLC2A2 (also called GLUT2)* rs5400-A allele (Ile110) [39,41], and we confirmed that participants carrying this A-allele had higher caries scores. We showed that two additional SNPs within the *SLC2A2* gene are also associated with caries susceptibility, however, all three SNPs displayed a high degree of genetic linkage (r^2^ > 0.93). None of the *SLC2A2* SNPs were associated with reported or preferred sweet food intake, or PT or TT for sweet in the present study, which could indicate that the effects of these SNPs on caries occur through a pathway which is independent of effects on sweet taste. Interestingly, SNP rs5400 (or SNP rs11920090, which is in perfect linkage disequilibrium) has been associated with risk of type 2 diabetes [42,43], total fasting serum cholesterol level [44], cardiovascular disease [45] and prostate cancer [46], conditions that have been suggested to correlate with higher caries risk [38]. 

In addition to confirming the previously known association between the *SLC2A2* rs5400 allele and caries, the present study found that allelic variation within the *SLC2A4* gene was associated with threshold levels for sweet taste (TT_sweet_), sweet food intake and caries status. *SLC2A4* encodes an additional glucose transporter (GLUT4) which is predominantly expressed in sweet sensing *TAS1R3* positive taste cells/buds [47]. In addition, expression of *SLC2A4* has been found in the acinar and ductal cells of rodent salivary glands and is suggested to have a role in glucose transportation from blood to saliva [48,49,50]. Increased levels of glucose in blood is associated with increased levels in saliva as well as increased caries risk [51,52]. Previous studies report that *SLC2A4* was expressed at lower levels in type 2 diabetic patients, and gene expression levels were associated with two SNPs (rs5417 and rs5418) in the 5 prime untranslated region of (5′UTR) [53], suggesting a relationship between *SLC2A4* 5′UTR genetic variants and gene expression. In line with these studies, we found that a variation in the *SLC2A4* 5′UTR, i.e., the rs5412, as well as the haploblock with rs5412, rs5415 and rs5418, was associated with caries status, suggesting *SLC2A4* as a potential candidate gene for caries risk via saliva glucose regulation.

In conclusion, the present results support the hypothesis that taste preferences for sweet, bitter and sour are partly genetically driven with effects on choices of foods and drinks and potential effects on diet-associated diseases though an overlap with caries was only found for rs5415 in the *SLC2A4* gene. Effects of several previously reported genetic variants on taste and diet preference were confirmed and a previously unreported association between allelic variation in *SLC2A4* and dental caries was identified. This is of high relevance to disentangle since even marginal risk factors may have a major worldwide effect on the overall incidence of highly prevalent and costly diseases, such as caries. Future research directions should aim at replicating the present findings in follow-up studies and expanding sample sizes to explore the effects of low-frequency genetic variation. Future research should also delineate the role of glucose transporter genes in dental caries per se but also as a shared risk factor with cardiometabolic diseases. 

## Figures and Tables

**Table 1:** Participant characteristics. Data are shown for all participants and when split by preferred concentration of sweet (PT_sweet_). Differences in subject distributions in the PT_sweet_ groups were tested with Chi^2^ test, and between means with Student’s *t*-test.

Factors	All Participants (*n* = 127)	Preferred Taste (PT)_sweet_ Groups		*p*-Value
		Low (*n* = 58)	High (*n* = 69)	
Male, Female, %	47.2, 52.8	37.9, 62.1	55.1, 44.9	0.054
Body Mass Index ^1^, kg/m^2^	23.0 (22.4, 23.6)	22.3 (21.4, 23.3)	23.6 (22.8, 24.3)	0.039
Smoking yes, %	4.7	3.4	5.8	0.687
Swedish snus yes, %	8.7	3.4	13.0	0.055
Diet intake ^1^				
Energy, kcal/day	1748 (1624, 1871)	1666 (1482, 1848)	1820 (1650, 1990)	0.226
Carbohydrate, E%	40.6 (39.3, 42.8)	40.9 (39.0. 42,8)	40.3 (38.5, 42.0)	0.626
Protein, E%	14.0 (13.4, 14.5)	13.7 (12.9, 14.5)	14.2 (13.4, 14.9)	0.445
Fat, E%	44.1 (42.8, 45.5)	44.3 (42.1, 45.9)	44.0 (42.1, 45.9)	0.808
Sucrose, E%	6.0 (5.6, 6.4)	5.9 (5.4, 6.5)	6.1 (5.6, 6.6)	0.652
Saliva flow ^1^, mL/min	1.5 (1.4, 1.6)	1.3 (1.1, 1.5)	1.7 (1.5, 1.8)	0.030
Number of teeth	27.5 (27.2, 27.7)	27.5 (27.2, 27.7)	27.5 (27.2, 27.7)	0.609
Caries status				
Caries affected ^2^, %	56.7	60.3	53.6	0.446
DeFS ^1^	4.4 (3.2, 5.6)	3.6 (2.4, 4.8)	5.0 (3.0, 7.0)	0.238

^1^ Values are mean (95% CI) and dietary variables are adjusted for sex. For other variables, adjustment for sex did not affect the results. E% for energy proportion from total energy intake. ^2^ “Caries affected” was defined as DeFS >0.

**Table 2:** Associations between SNPs, sweet, sour and bitter taste threshold (TT) and preference (PT) and food intake. Generalized linear modelling was used to evaluate gene polymorphisms in relation to the dependent phenotype variables. All models included sex as covariates. All tests were controlled by the Benjamini and Hochberg procedure, and models where the *p*-value passed multiple correction using Benjamini and Hochberg false discovery rate of 0.05 are presented. A full list of model results are presented in Tables S3–S5. Variants with minor allele count <5 were excluded. Mean (95% CI) values for the phenotypes are given for each genotype group.

Pheno-Type	Gene	rs-Number	Minor/Major	Genotype Group 1 vs. 2	Freq. of Geno-Type Group 1	Test Group Mean (95% CI)		*p*-Value
						Group 1	Group 2	
TT_sweet_	*GNAT3*	rs17260734	T/A	TT + TA vs. AA	69.4%	3.1 (2.9, 3.2)	2.6 (2.4, 2.8)	0.0007
	*GNAT3*	rs7792845	T/C	TT + TC vs. CC	57.3%	3.1 (2.9, 3.3)	2.7 (2.5, 2.8)	0.0002
	*SLC2A4*	rs2654185	A/C	AA + AC vs. CC	49.6%	3.1 (2.9, 3.4)	2.7 (2.6, 2.9)	0.0016
	*SLC2A4*	rs5415	T/C	TT + TC vs. CC	43.5%	3.2 (3.0, 3.4)	2.7 (2.6, 2.8)	0.0004
PT_sweet_	*TAS1R1*	rs4908923	G/A	GG + GA vs. AA	18.1%	4.4 (4.1, 4.8)	5.5 (5.0, 6.0)	0.0010
	*TAS1R2*	rs9988418	T/C	TT + TC vs. CC	3.9%	2.6 (1.3, 3.8)	4.7 (4.4, 5.0)	0.0010
	*TAS1R2*	rs28652778	T/C	TT vs. CT + CC	5.6%	5.8 (5.1, 6.5)	4.6 (4.3, 4.9)	0.0013
	*GNAT3*	rs7792845	T/C	TT vs. CT + CC	14.5%	5.7 (5.0, 6.3)	4.5 (4.1, 4.8)	0.0011
TT_sour_	*TAS1R2*	rs12035074	C/G	CC + CG vs. GG	44.4%	3.0 (2.8, 3.2)	3.4 (3.3, 3.6)	0.003
	*GNAT3*	rs6947745	T/C	TT vs. TG + GG	5.5%	3.0 (3.0, 3.1)	3.3 (3.2, 3.5)	0.0004
PT_sour_	*TAS1R1*	rs4908932	T/G	TT vs. TG + GG	5.6%	5.2 (4.8, 5.5)	4.4 (4.1, 4.7)	0.0002
	*TAS1R2*	rs12035074	C/G	CC + CG vs. GG	44.4%	3.9 (3.5, 4.3)	4.8 (4.5, 5.2)	0.0003
	*TAS1R2*	rs35874116	C/T	CC vs. CT + TT	8.7%	5.4 (4.7, 6.1)	4.3 (4.0, 4.6)	0.0018
TT_bitter_	*TAS2R50*	rs2218820	T/C	TT vs. TC + CC	21.3%	2.6 (2.4, 2.9)	2.2 (2.1, 2.3)	0.002
Sweet	*TAS1R2*	rs9701796	G/C	GG + GC vs. CC	38.4%	0.9 (0.8, 1.0)	1.3 (1.1, 1.5)	0.003
food	*SLC2A4*	rs2654185	A/C	AA vs. CA + CC	8.0%	0.7 (0.4, 1,0)	1.2 (1.0, 1.3)	0.002
intake	*SLC2A4*	rs5418	G/A	GG + GA vs. AA	11.2%	0.7 (0.5, 0.9)	1.2 (1.0, 1.3)	4.8 × 10^−5^
	*TAS1R2*	rs28374389	C/T	CC < CT < TT	7.1%	-	-	0.003

**Table 3:** Spearman correlation coefficients between taste recognition threshold (TT) and preference (PT) and food preference scores or intake frequencies in sweet, bitter, sour taste categories, as well as food items and nutrients associated with any of the three taste categories. P-values are shown in superscript. A full list of correlations is presented in Table S6.

	TT_sweet_	PT_sweet_	TT_bitter_	PT_bitter_	TT_sour_	PT_sour_
**Food taste clusters**						
Food preference for sweet foods	0.13^0.173^	0.36^<0.001^				
Intake of sugary foods	0.18^0.044^	0.22^0.014^	−0.24^0.008^			
Food preference for bitter foods				0.14**^0.153^**		
Intake of bitter foods				0.15^0.092^		
Food preference for sour foods				−0.22^0.019^	−0.10^0.301^	0.17^0.060^
Intake of sour foods					−0.27^0.003^	
**Foods items**						
Intake of sweets	0.20^0.025^		−0.19^0.037^			
Intake of juice	0.21^0.022^					
Food preference for ice cream		0.19^0.039^				
Food preference for sweet rolls/rusk		0.24^0.010^				
Food preference for raisins		0.24^0.008^				
Intake of syrups		0.22^0.012^	−0.23^0.010^			
Food preference for non-fermented milk		0.26^0.004^				
Intake of non-fermented milk		0.21^0.020^				
Intake of pancake			−0.18^0.048^			
Food preference for Brussels sprouts				0.19^0.044^		
Intake of filter brewed coffee				0.15^0.088^		
Intake of boiled coffee				0.18^0.042^		
Intake of beer		0.20^0.026^		0.18^0.046^		
Food preference for juice				−0.23^0.012^		
Intake of cookies/cakes				0.21^0.018^		
Intake of apples, pears, peaches					−0.29^0.001^	
Food preference for lemon						0.20^0.027^
**Nutrients**						
Disaccharides, g/day		0.24^0.008^				
Sucrose, g/day		0.17^0.056^	−0.22^0.014^			
Ascorbic acid, mg/day					−0.21^0.018^	

**Table 4:** Sweet taste and sugar intake associated genetic polymorphisms and caries status. Associations were tested in generalized linear models with total number of decayed and filled tooth surfaces (DeFS) as dependent variable and each of the SNPs as predictors. All models included tooth brushing frequency as covariate. All tests were controlled by the Benjamini and Hochberg procedure, and models where the *p*-value passed multiple correction using Benjamini and Hochberg false discovery rate of 0.05 are presented. Variants with minor allele count <5 were excluded. Mean (95% CI) values for the phenotype are given for each genotype group.

Gene	Rs-Number	Minor/Major	Genotype Group 1 vs. 2	Freq. of Geno-type Group 1	DeFS (Group Mean (95% CI))		*p*-Value
					Group 1	Group 2	
*GNAT3*	rs6962693	G/T	GG + GT vs. TT	15.7%	1.2 (0.5, 2.8)	4.5 (3.5, 5.8)	2.0 × 10^−3^
*TAS1R1*	rs4908932	T/G	TT + TG vs. GG	33.1%	6.4 (4.8, 8.6)	1.9 (1.0, 3.6)	1.7 × 10^−5^
*TAS1R2*	rs6685177	A/G	AA vs. AG + GG	7.1%	8.8 (6.2, 12.4)	3.1 (2.0, 4.8)	4.5 × 10^−5^
	rs28374389	C/T	CC vs. CT + TT	7.1%	8.8 (6.2, 12.4)	3.1 (2.0, 4.8)	4.5 × 10^−5^
	rs28410948	C/T	CC vs. CT + TT	10.2%	8.0 (5.9, 11.0)	2.9 (1.7, 4.8)	2.0 × 10^−4^
*SLC2A2*	rs1996220	G/A	GG + GA vs. AA	26.0%	7.3 (5.4, 9.7)	2.1 (1.2, 3.7)	2.0 × 10^−6^
	rs5400	A/G	AA + AG vs. GG	26.0%	7.3 (5.4, 9.7)	2.1 (1.2, 3.7)	2.0 × 10^−6^
	rs11917504	T/A	TT + TA vs. AA	23.6%	7.8 (5.8, 10.4)	2.2 (1.3, 3.7)	2.3 × 10^−7^
*SLC2A4*	rs5415	T/C	TT vs. TC + CC	6.6%	8.8 (4.9, 15.9)	3.2 (2.3, 4.5)	3.4 × 10^−3^

## Supplementary Materials

The following is available online at https://www.mdpi.com/2072-6643/11/7/1491/s1, Table S1: Summary of selected references for choosing genes used in this study, Table S2: Single Nucleotide Polymorphisms genotyped in the study participants, Table S3: Associations between SNPs, sweet taste threshold (TT), sweet taste preference (PT), sweet taste food intake and caries. Generalized linear modelling (GLM) was used to evaluate gene polymorphisms in relation to the dependent phenotype variables. All models included sex as covariates. Variants with minor allele count <5 are marked in red. Models where the p-value passed multiple correction using Benjamini and Hochberg false discovery rate of 0.05 and allele count >5 are marked in bold letters, Table S4: Associations between SNPs, sour taste threshold (TT), sour taste preference (PT) and sour food intake. Generalized linear modelling was used to evaluate gene polymorphisms in relation to the dependent phenotype variables. All models included sex as covariates. Variants with minor allele count <5 are marked in red. Models where the p-value passed multiple correction using Benjamini and Hochberg false discovery rate of 0.05 and allele count >5 are marked in bold letters, Table S5: Associations between SNPs, bitter taste threshold (TT), bitter taste preference (PT) and bitter food intake. Generalized linear modelling was used to evaluate gene polymorphisms in relation to the dependent phenotype variables. All models included sex as covariates. Variants with minor allele count <5 are marked in red. Models where the *p*-value passed multiple correction using Benjamini and Hochberg false discovery rate of 0.05 and allele count >5 are marked in bold letters, Table S6: Spearman correlation coefficients, with associated *p*-values, between taste recognition threshold (TT) and preference (PT) and food preference scores or intake frequencies in taste groups as well as all food items and some assessed nutrients. Figures in bold are also shown in Table 3 in the main document.
